# N-Terminal *Plasmodium vivax* Merozoite Surface Protein-1, a Potential Subunit for Malaria Vivax Vaccine

**DOI:** 10.1155/2013/965841

**Published:** 2013-09-28

**Authors:** Fernanda G. Versiani, Maria E. Almeida, Luis A. Mariuba, Patricia P. Orlandi, Paulo A. Nogueira

**Affiliations:** ^1^Universidade Federal do Amazonas, Avenida General Rodrigo Octávio Jordão Ramos 3000, Campus Universitário, Coroado I, 69077-000 Manaus, AM, Brazil; ^2^Instituto Leônidas e Maria Deane-Fiocruz, Rua Teresina 476, 69057-070 Manaus, AM, Brazil

## Abstract

The human malaria is widely distributed in the Middle East, Asia, the western Pacific, and Central and South America. *Plasmodium vivax* started to have the attention of many researchers since it is causing diseases to millions of people and several reports of severe malaria cases have been noticed in the last few years. The lack of *in vitro* cultures for *P. vivax* represents a major delay in developing a functional malaria vaccine. One of the major candidates to antimalarial vaccine is the merozoite surface protein-1 (MSP1), which is expressed abundantly on the merozoite surface and capable of activating the host protective immunity. Studies have shown that MSP-1 possesses highly immunogenic fragments, capable of generating immune response and protection in natural infection in endemic regions. This paper shows humoral immune response to different proteins of PvMSP1 and the statement of N-terminal to be added to the list of potential candidates for malaria vivax vaccine.

## 1. Introduction

 Malaria remains one major infectious disease that affects most tropical regions of the world. Since the 1980s, the world efforts for the development of vaccines were exclusively directed for the most virulent malaria species (*Plasmodium falciparum*), *P. vivax* produces around 80 to 300 million clinical cases per year, and several reports of severe malaria cases have been noticed in the last few years [1–5] by similar complications and pathogenic mechanisms frequently observed in malaria caused by *P. falciparum* [6, 7]. Drug resistance of *P. vivax* to commonly used antimalarial drugs has also been reported worldwide [1, 3, 8–16]. Therefore, currently, the designation “malaria benign” is considered as a mistake [17] challenging the current view of *P. vivax* as a less harmful parasite and warranting development of an effective vaccine.

Although the current investments are focused on orthologs of *P. falciparum*, developing a vaccine for *P. vivax* represents a major challenge especially considering the lack of *in vitro* cultures and hence delays in identifying and developing antigenic subunits for a functional malaria vaccine.

In hopes of reducing morbidity and mortality caused by *P. vivax* infection, the most important malaria vaccine candidates are outlined in the preerythrocytic stage: CSP (*Circumsporozoite protein*) and SSP2/TRAP (*Thrombospondin related anonymous protein*); asexual erythrocytic cycle: the subunits of MSP-1 (42 kDa, 19 kDa, and a precursor molecule of 200 kDa), MSP-9, DBP-RII (*Duffy binding protein receptor*-2), and AMA-1 (*apical membrane antigen*-1), and the antigens of the sexual erythrocytic cycle, Pvs25 and Pvs28. Other genes of *Plasmodium vivax* asexual erythrocytic cycle that have been identified as potential vaccine candidates are the PvMSP-3 family (*pvmsp3a/3b/3g*): PvMSP-4, PvMSP-5 and PvRBP-5 1/2 [18]. 

However, one of the major difficulties in developing a malaria vaccine is the genetic diversity of highly polymorphic surface antigens of *P. falciparum* and *P. vivax* in different geographic areas around the world. The problem of polymorphism tends to be more important for vaccines based on blood stage targets of naturally acquired immunity [19, 20].

## 2. A Target for Production of Vaccine

An effective vaccine against malaria has long been envisaged as a valuable addition to the available tools for malaria control. Nonetheless, extensive polymorphism and antigenic variation of key parasite proteins hamper the development of an effective vaccine to generate a long-lasting protection. Unfortunately for many years, studies on the species *Plasmodium vivax* were neglected and as a consequence only few antigens were focus, such as Duffy binding protein (DBP) and circumsporozoite protein (CSP) [2]. The development of resistance in *P. vivax* to several first-line antimalarial drugs and severity of disease have been driven in a *P. vivax* vaccine [21].

Availability of the *P. vivax* genome has contributed to antigen discovery, but new means to test vaccines in future trials remain to be designed. In addition to the CSP, much effort has been spent on other candidates in preclinical studies such as: the sporozoite surface protein 2 (SSP2/TRAP); smaller fragments (i.e., the 19 and 42 kDa domains) and whole of *P. vivax* MSP1 fragments (Pv200L); the parasite ligand that contains the receptor-binding domain for Duffy binding protein (DBP-RII); the apical membrane antigen 1 (AMA-1); the ookinete surface antigens (Pvs25 and Pvs28) candidate to transmission blocking vaccines; and several other genes from the asexual blood stages (e.g., MSP3, MSP4, MSP5, MSP9, and the reticulocyte binding protein) that have been identified and partially characterized [17, 18, 21].

The merozoite stage disrupts the erythrocytes and infects again new erythrocytes in the bloodstream. MSP1 protein demonstrated to have important role in the induction of protective immune response in the test conducted using the species *P. chabaudi* in challenge of infection in mice. The study showed that the gene of MSP1 dominates the species-specific protective immunity [22].

Individuals exposed to malaria develop immunity that protects from the clinical manifestations of infection [23]. Based on this, efforts to produce vaccine have been directed to the major merozoite antigen, the MSP1 complex. 

During the invasion to erythrocytes, the processing of precursor MSP-1 leads to production of four fragments of 82, 30, 38, and 42 kDa. The 42 kDa C-terminal fragment is further cleaved into two fragments a 33 and 19 kDa, the latter being the only remaining bound to the membrane, apparently anchored via GPI. It has been described that proteolytic processing of ortholog *P. vivax* MSP-1 is similar to that of *P. falciparum*, which is necessary for parasite invasion of erythrocytes. Recently, it was demonstrated that a region of the PvMSP1_19_ might be an essential parasite adhesion molecule in the *P. vivax* merozoite binding to erythrocytes [24]. 

These *P. vivax* MSP-1 fragments (MSP1_19_ and MSP1_42_) displayed strong immunogenicity in BALB/c mice and in *Aotus* monkeys. In immunization experiments in mice with formulations containing MSP1_19_ and MSP1_42_ fragments using CFA as an adjuvant, aluminum and Montanide ISA 720 achieved better results with the highest levels of antibodies. The pattern of IgG isotype was IgG1 with higher titles, followed by IgG3 and IgG2 for both proteins [25]. 

Different recombinant proteins representing the MSP1(19) of *Plasmodium vivax* were constructed with addition of the T-cell Pan-allelic DR epitope (His6MSP1_19_-PADRE) or transformed in the presence of a TLR5 agonist (Toll-like receptors) from *Salmonella typhimurium* as an adjuvant. As a result, a strong adaptive immune response and long duration were gained in vaccinated mice [26, 27]. 

Another adjuvant tested was the gold nanoparticle (GNP) used as a carrier of peptides, drugs, and others to target tissues. According to tests conducted by Parween et al. [28], there was production of antibodies in mice immunized with the PvMSP1_19_ and PfMSP1_19_ fragments using GNP and aluminum as an adjuvant. The adjuvant caused an increase of immunogenicity when compared to aluminum and to GNP alone.

The immunogenicity of recombinant PvMSP1_19_ expressed in yeast containing two epitopes of tetanus toxin in a formulation containing aluminum and copolymer P1005 was found in *Saimiri* monkeys with an increase of IgG antibodies after three immunizations and decrease in parasitaemia after being challenged by infection of *P. vivax* [29].

Rhesus monkeys previously immunized with formulations based on PvMSP1_42_ had significantly lower parasite burdens after challenge with *Plasmodium cynomolgi* (a phylogenetic species related to *P. vivax*) when compared to a control group that received only adjuvant [30].

Primates immunized with a formulation containing a fragment of the N-terminal extremity of MSP-1 called pv200L in Montanide ISA 720 showed vigorous antibody responses that recognized parasite antigens in western blot and IFAT assays. Immunized animals were partially protected against *P. vivax* challenge, as determined by lower parasitemias, reduced anemia, and spontaneous parasite clearance [31]. 

Studies on *P. falciparum* MSP1 as vaccines may provide knowledge for the development of vaccines for *P. vivax* [32–35]. That Phase I studies evaluated two formulations of vaccines containing the portion PfMSP1_42_ have shown safety tolerability, and detectable antibodies of 74% and 81% of individuals after 3 vaccinations. Although the protection was species-specific, they were not immunogenic enough to obtain a significant biological effect when performed in *in vitro* test [36]. Phase 1B studies using other formulations obtained immunogenicity, safety, and tolerability when tested in children [37]. 

Development of an efficacious *P. falciparum* vaccine will create optimism toward the likelihood of developing a multispecies vaccine. In addition, a growing number of antigens are being added to the list of potential candidates and new formulations must be tested to increase the immunogenicity of vaccines using new adjuvants.

## 3. MSP1 Complex

In ultrastructural studies, the surface of the merozoite appears as a thick fibrillar coat composed of integral and peripheral membrane proteins that have generally been termed merozoite surface proteins (MSPs). It is thought that MSPs are the important components in the erythrocyte invasion mediating the relatively weak and reversible initial interactions between the parasite and RBC [43–45]. The *Plasmodium* merozoite antigens have been proposed as targets for blood stage vaccines [18].

Amongst this family of molecules, the MSP1 has been studied intensively because of the need to understand the mechanisms involved in erythrocyte invasion by parasites and its importance as an antigen capable of activating the host protective immunity [39, 46]. MSP1 is a polymorphic protein expressed abundantly on the merozoite surface and is part of a complex in which proteins are associated with MSP6 and MSP7, which bind to merozoite surface anchor by glycosylphosphatidylinositol (GPI) and mediate initial interactions between the parasite and the erythrocyte [44]. 

The ortholog of *Plasmodium falciparum* MSP1 complex that contains two other parasite proteins, MSP6 and MSP7, is likely to be an important component in the erythrocyte invasion and it has been the focus of much research as a vaccine candidate [44]. This complex undergoes two processes cleavages, where the first processing performed by the protease subtilisin-like (SUB1) [43] generates four fragments of approximately 83, 30, 38, and 42 kDa, known as, MSP1_83_, MSP1_30_, MSP1_38_, and MSP1_42_ [46, 47].

 MSP1 is a glycoprotein abundant on the surface and essential for merozoite development due to its involvement in erythrocyte invasion [47]. The gene described in *Plasmodium falciparum* MSP1 (PfMSP1) encodes a protein of 190 kDa divided into 17 blocks classified as conserved, semiconserved, and variables [48]. Many studies have used the polymorphic regions of MSP1 as genetic markers to determine the genetic diversity. Based on Block 2, PfMSP1 allelic variants fall under three major types: MAD-20, K1, and RO33, but their frequency varies in different geographical areas, even in neighboring villages [49]. 

**Figure 1:**
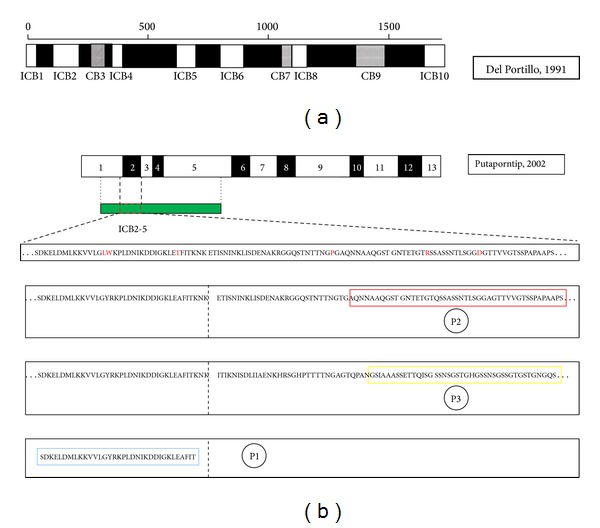
Schematic representation of the protein PvMSP1 according to two classifications adapted from Bastos et al. [38]. (a) Diagram showing the division of PvMSP-1 into interspecies conserved blocks (ICBs) among *P. falciparum*, *P. vivax*, and *P. yoelii* (blank), blocks conserved between *P. falciparum* and *P. vivax* (in gray), and polymorphic blocks (black), by Del Portillo et al. [39]. Another scheme showing the division of PvMSP-1 in 13 blocks, 7 conserved blocks (blank), and six polymorphic blocks (black) by Putaporntip et al. [40]. (b) Location and amine acids sequences of three recombinant proteins correspondent to Block-2 variants of PvMSP-1; (P1) conserved region located upstream of Block2; (P2) corresponding to the repeats of haplotype *belem*; and (P3) corresponding to the repeats of another haplotype of Pv-MSP1 Block 2 circulating in Manaus [41].

The primary structure of ortholog of (200-kDa)* Plasmodium vivax *MSP1 glycoprotein (PvMSP1) was based on two studies [39, 40] (Figure 1(a)). At first, the original division was represented in interspecies conserved blocks (ICB) by comparison of the MSP1 of *P. vivax* (strain isolated from *belem*) between MSP-1 of *P. falciparum*, *P. vivax*, and *P. yoelii*. According to this, PvMSP-1 consisted of seven highest amino acid similarities between MSP-1 of the species *P. falciparum*, *P. vivax*, and *P. yoelii* (ICB1, ICB2, ICB4, ICB5, ICB6, ICB8, and ICB10). In three regions calling them CB conserved blocks, amino acid similarity was even higher among *P. falciparum*, *P. vivax* (CB3, CB7, and CB9), and also extensions called polymorphic blocks, where the amino acid similarity was less than 45% [39]. 

The second was based only on sequences of *Plasmodium vivax*, and currently accepted *pvmsp1* gene consists of 13 blocks, being seven conserved blocks (blocks 1, 3, 5, 7, 9, 11 and 13) flanked by six variable blocks called polymorphic blocks (2, 4, 6, 8, 10 and 12) [40]. The extensive sequence divergence in variable domains of orthologs *msp1* has been maintained by balanced selection over five million years, most likely as a result of variant-specific immune pressure [50].

## 4. Advantage of Studying the N-Terminus MSP1 as Candidate Vaccine

A large body of evidence indicates that MSP1 is highly immunogenic in natural malarial infections and often associated with parasite exposure. Humoral immune response against PvMSP1 has been shown to be mostly against the polymorphic domains [51–56]. Using recombinant proteins representing different portions of PvMSP1, several studies showed that conserved regions are weakly immunogenic and, contrariwise increasing natural humoral response was observed when stretches of amino acids of polymorphic blocks were constructed between the ICBs [51, 53, 57]. The serological surveys conducted in different regions of Brazilian Amazon (Amazonas, Rondonia, and Belem) showed high prevalence of IgG to ICB2-5 and denominated N-terminus of the PvMSP-1 [42, 51, 54, 58]. Seroepidemiological studies conducted in various tropical areas of the world also showed higher levels of IgG against N-terminal PvMSP1 in individuals previously exposed to the parasite or in *P. vivax*-infected patients [42, 53, 55, 56, 58].

Despite that recombinant ICB2-5 contains long stretches of amino acids that are conserved among the *P. vivax* haplotypes (*Belem* and *Salvador*), it has been well-established that these antibodies primarily recognize the variable domains (Blocks 2 and 4) of ICB2-5 [38, 39]. According to Valderrama-Aguirre [31], acquisition of natural IgG antibodies against a panel of allelic variant proteins of major polymorphic blocks of PvMSP1 showed that the Block 2 was poorly immunogenic although it had been the most recognized by subjects exposed to malaria in western Brazilian Amazon indicating that the acquisition of variant-specific response requires successive boosting of these polymorphic blocks [38]. 

In a prospective 1-year longitudinal study of a human population in the Brazilian Amazon Basin in which asymptomatic *P. vivax* patients had been previously, identified high levels of antibodies against the N-terminus of Pv-MSP1 were observed in individuals clinically protected from malaria. This was the first study to demonstrate an association of clinical protection and reduced risk of infection with naturally acquired IgG antibodies against a *P. vivax* antigen, PvMSP1 [54]. 

In another study, three hundred thirteen residents of the Rio Pardo rural settlement (Amazonas State, Brazil) were evaluated in a cross-sectional and longitudinal follow-up over two months (on site), wherein thick blood smear and rRNA gene-based nested real-time PCR were used to discriminate symptomless *Plasmodium vivax*-infected individuals who did not develop clinical symptoms during two months [42]. Assessing the acquired immune response against N-terminus PvMSP1, levels of IgG3 anti-ICB2-5 were higher in symptomless in *Plasmodium vivax* infected individuals than those of subjects who had acute malaria or those uninfected ones, raising importance of the N-terminus PvMSP1 to the rationale of malaria vaccine designs [42]. 

**Figure 2:**
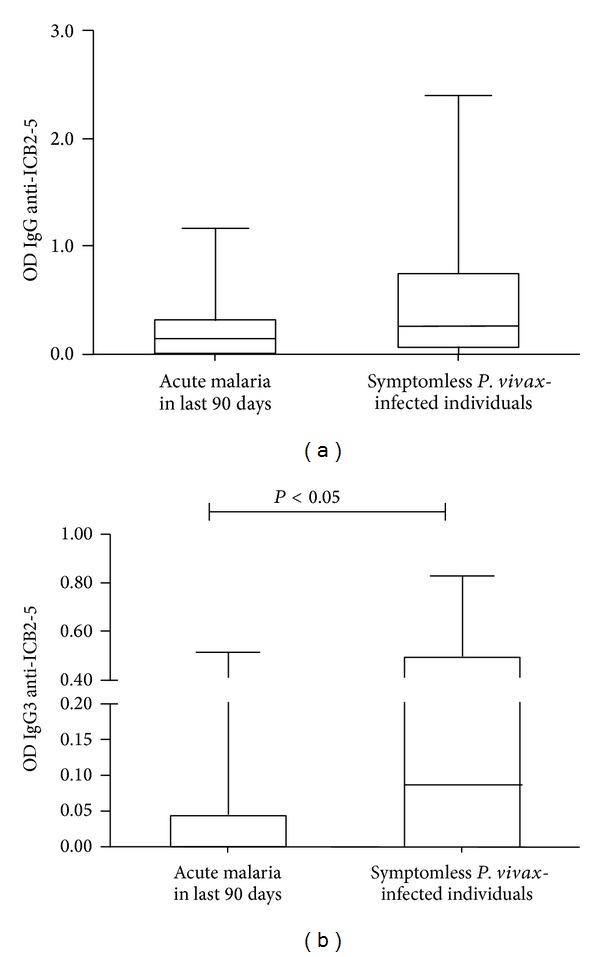
Comparison of total IgG and IgG3 levels against ICB2-5. Comparison of antibodies of individuals from study [42] separated according to subjects who have had acute malaria in last 90 days and symptomless *P. vivax*-infected individuals. Median of antibodies against ICB2-5 was compared by Kruskal Wallis. (a) Total IgG; (b) IgG3.

To assess the acquisition of antibodies against ICB2-5 after an episode of acute malaria, a retrospective search was performed on SIVEP-malaria database. Total IgG levels of individuals who had an acute malaria ninety days before one cross-sectional follow-up were not different from symptomless *P. vivax*-infected individuals (Figure 2(a)). However, IgG3 anti ICB2-5 levels were very low (*P* < 0.05) in individuals who have had malaria in the last 90 days corroborating the idea of poor immunogenicity of Block 2 and requirement of consecutive boosting for variant-specific humoral response (Figure 2(b)). 

When a panel of recombinant proteins of three Block 2 variants of PvMSP1 (Figure 1(b)) was tested against symptomatic versus asymptomatic sera, IgG3 from symptomless *P. vivax*-infected individuals reacted against most of the Block-2 variants of PvMSP-1 and with higher levels of antibodies. The results indicated prevalence of subclasses IgG reducing the effect of genetic diversity of PvMSP1 [41]. 

**Figure 3:**
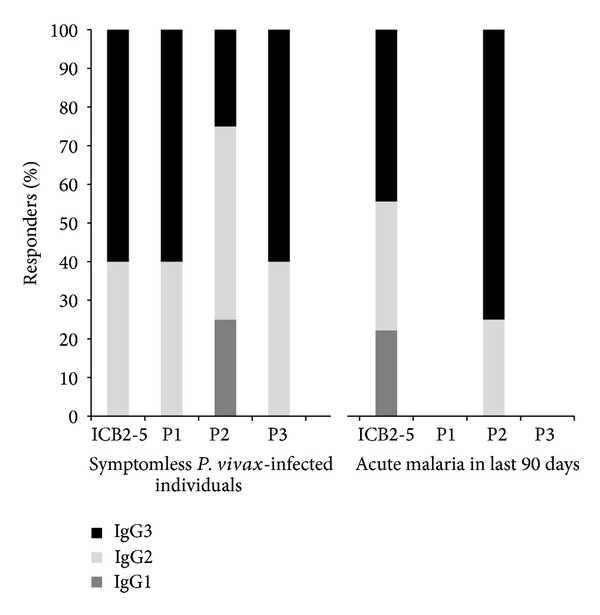
Frequencies of subclasses IgG responders agains a panel of variants of PvMSP1 Block 2. Percentages of subclasses responders agains ICB2-5 and a panel of major allelic variants of Block 2 expressed as recombinant proteins are shown between subjects who have had malaria in last 90 days and symptomless *P. vivax*-infected individuals from Rio Pardo, an agricultural settlement of Rio Pardo, Presidente Figueiredo municipality, northeast region of Amazonas State, Brazil [42].

Accomplishing retrospective study, individuals who had malaria in the last 90 days showed a restricted humoral response to the same panel of proteins (Figure 3). In addition, the predominance of IgG3 against most of the Block-2 variants of PvMSP-1 confirms findings that these antibodies were induced against repetitive polymorphic sequences [42].

## 5. Prospects for the Future

The potentiality of ICB2-5 as a potential subunit candidate for malaria vaccine development was based on findings that high levels of IgG3 antibodies against the N-terminus of Pv-MSP1 in asymptomatic individuals infected with *Plasmodium vivax* were associated with clinical protection and reduced risk of infection with *Plasmodium vivax* [42, 54]. 

Similarly, significant persistence of effective antibodies of IgG3 anti N-terminal of *P. falciparum* MSP1 was associated with prolongation time without malaria [59]. According to the authors, the presence of IgG3 anti *P. falciparum* MSP1 Block 2 of the individuals who would be held by asymptomatic infection confers a protective effect for extended periods. As such, it has considerable potential as a candidate target for vaccine design and/or clinical trials [60, 61].

These studies allow us to conclude that for the progress of a vaccine for malaria, a deeper understanding repertoire of variable domains of candidates and demand strategies to sustain the levels of specific IgG3 regularly may be a new issue.
